# Korean medicines for poor ovarian reserve in infertility: A protocol for a multicenter observational study

**DOI:** 10.1097/MD.0000000000017731

**Published:** 2019-11-01

**Authors:** Tae-Young Choi, Ji Hee Jun, Hye Won Lee, Lin Ang, Eun Seop Kim, Ho Yeon Go, Sooseong You, Myeong Soo Lee

**Affiliations:** a Clinical Medicine Division; b Herbal Medicine Research Division, Korea Institute of Oriental Medicine; c You and Green Korean Medical Clinic, Daejeon; d Internal Medicine, College of Korean Medicine, Semyung University, Chungju, South Korea.

**Keywords:** acupuncture, female infertility, herbal medicine, Korean medicine, poor ovarian reserve, prospective observational study

## Abstract

**Introduction:**

Many infertile patients have used Korean medicines (KMs) as a primary or adjuvant therapeutic method to improve in vitro fertilization success rates. The aim of this multicenter observational study is to investigate the effects of KMs on poor ovarian reserve (POR) in infertile patients.

**Methods and analysis:**

We will perform a prospective multicenter observational study. This study will recruit 50 women between 25 and 44 years of age with infertility caused by POR from among patients who visit the KM clinic. All participants will visit the KM clinic on the 2nd or 3rd day of menstruation to receive the KMs. KM treatment will be delivered for 3 menstrual cycles (3 months) and will include herbal decoction, acupuncture, or moxibustion on demand. Every participant will be assessed based on KM clinical symptoms, a quality of life questionnaire, and ovarian reserve test results.

**Ethics and dissemination:**

The study was approved by Institutional Review Board of Semyung University (SMU-IM-190501). The results will be published in a peer-reviewed journal and will be disseminated electronically and in print.

**Trial registration number:**

Clinical Research Information Service: KCT0004209.

## Abstract 2

Supplemental Digital Content is available in the text

## 1 Introduction

Current fertility rates are well below those needed for a population replacement rate of 2.1 in most Organization for European Economy Cooperation (OECD) countries; however, South Korea in particular has the lowest fertility rate among all the OECD countries.^[1]^ The high infertility rate of the country is thought to be partially attributable to social factors such as the increase in the number of working couples, marriage age, stressors, and environmental pollution.^[2]^ While the government increasingly supports infertility treatments, such as in vitro fertilization (IVF), the fertility rate remains low.^[3]^

Ovulation disorders (prevalence rate, 15%) and tubal and intra-abdominal abnormalities (prevalence rate, 30–40%) are thought to be the causes of female infertility, but other causes are unknown in Western medicine.^[4]^ Patients with degraded ovarian function comprise 9% to 24% of all infertility patients, and the pregnancy rate after IVF and embryo transfer is as low as 30% to 40%.^[5]^ The largest obstacle in increasing the overall pregnancy rate is the low post-IVF pregnancy rate in patients with aggravated ovarian function who show a weak response to controlled ovarian hyperstimulation.^[6]^ Despite the many proposed ovulation induction methods for the low response group, no clinically significant differences have been reported.^[7]^ In addition, indiscreet assisted reproductive technology in Western medicine causes infertility patients to have a decreased quality of life and to incur high costs; moreover, artificial ovulation induction results in various side effects. Patients have been looking for complementary options to improve fertility.

Korean medicine (KM) has become very common as a primary or adjuvant therapy method for infertile patients^[3]^ and has been reported to improve IVF success rates.^[8]^ Approximately 60% of infertile patients had used KM treatment before using assisted reproductive technology in 2012.^[3]^ A study showed that KM improved ovarian quality; however, no regimen is available for infertility patients with deterioration of ovarian function.^[9]^ One systematic review reported that acupuncture and herbal medicine treatments had significantly improved ovarian function compared with Western medicine treatments for patients with deterioration of ovarian function.^[10]^ However, few studies have investigated the effects of KM for improving ovarian function in patients with deteriorating ovarian function. Therefore, we will perform a prospective multicenter observational study that reflects actual clinical practices including the interventions applied to patients and progress status.

## 2 Participants and methods

### 2.1 Study aims

The aim of this study is to investigate the effects of KM on poor ovarian reserve (POR) in infertility patients.

### 2.2 Study design/setting

**Figure 1:**
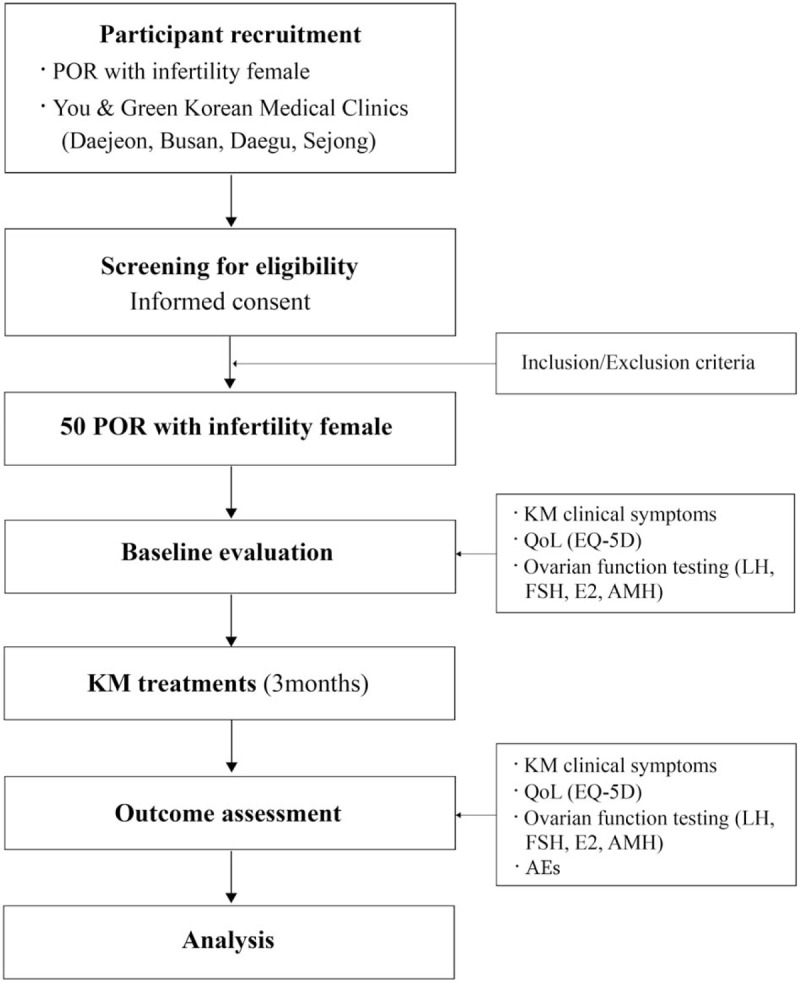
A flowchart of the study. AEs = adverse events, AMH = anti-Mullerian hormone, E2 = estradiol, EQ-5D = European Quality of Life-5 Dimensions, FSH = follicle-stimulating hormone, KM = Korean medicine, LH = luteinizing hormone, QoL = quality of life, POR = poor ovarian reserve.

This study is a prospective multicenter observational study. We will recruit patients from the You & Green Korean Medical Clinic in 4 regions (Daejeon, Busan, Daegu, Sejong) (Fig. 1). Data collection and follow-up schedules are shown in Table 1.

**Table 1:**
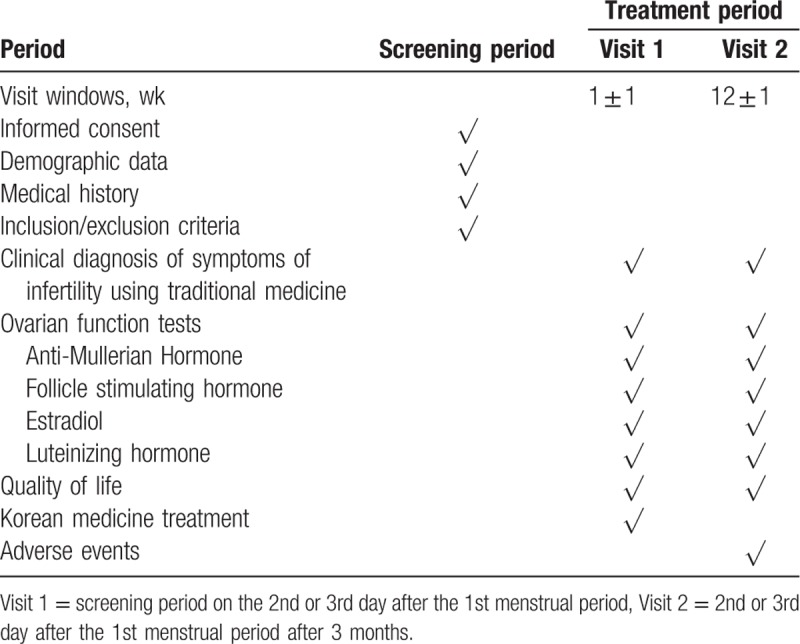
Schedule of study visits and assessments.

### 2.3 Study registration

This study is registered with the Clinical Research Information Service (https://cris.nih.go.kr/cris/en/): KCT0004209. Current protocol version is 1.1.

### 2.4 Eligibility criteria

#### 2.4.1 Inclusion criteria

We will include patients who are eligible according to the following criteria:

1. Females between 25 and 44 years of age who have been diagnosed with infertility.
2. Those without a history of surgical procedures but with an antral follicle count (AFC) < 4 or an anti-Müllerian hormone (AMH) level < 1.0 ng/mL in both ovaries confirmed through ultrasonographic examination.
3. Those with a history of surgical procedures but with an AFC < 4 or an AMH level < 1.0 ng/mL in both ovaries confirmed using ultrasonography.^[11]^
4. Failure to achieve a clinical pregnancy after 12 months or more (6 months for females aged 35 and above) of regular unprotected sexual intercourse.^[12]^
5. Written informed consent to participate in the study.
6. Compliance with the study regulations.

#### 2.4.2 Exclusion criteria

1. Those with an irregular menstrual cycle of less than 21 days or more than 40 days.
2. Those who take antidepressants, anti-serotonin, antipsychotics, or other anti-psychiatric drugs.
3. Those who received hormone therapy within 6 months.
4. Those who participated in other clinical trials within the past 6 months.

### 2.5 Recruitment

We will recruit 50 female patients with infertility at the You & Green Korean Medical Clinic. The researcher will explain the aim of this study and the details of the procedures and will obtain informed consent from potential subjects prior to the collection of information. Participants will be free to withdraw at any time during the study, and this will not affect their clinical treatment.

### 2.6 Intervention

The participants will be treated for 3 months with the standard protocol practiced at the You & Green Korean Medical Clinics. KM interventions (herbal medicine, acupuncture, electroacupuncture, herbal acupuncture, auricular acupuncture, moxibustion, manual therapy, etc) will be prescribed for infertility treatment in the same manner as in actual clinical practice, without the limitation of the number of interventional treatments. Herbal medicine will be prescribed according to individual symptoms and pattern identification. The detailed herbal prescriptions according to pattern identification are listed in Table 2. The dosages and contents of the herbs will be the same as that used in actual clinical practice. If necessary, acupuncture or another KM will be prescribed once a week. If the patient becomes pregnant during treatment, these treatments will be stopped.

**Table 2:**
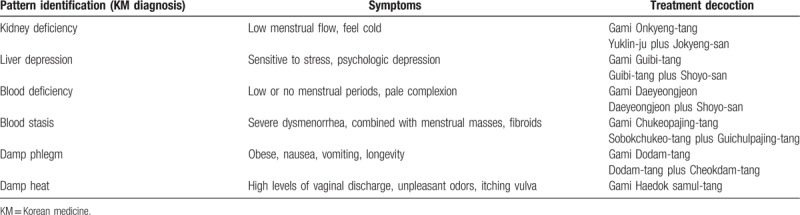
KM herbal prescription for KM diagnosis.

### 2.7 Outcomes measures

#### 2.7.1 Primary outcome

KM clinical symptoms questionnaire for pattern identification in gynecology: This questionnaire was developed for diagnosis pattern identification and measuring the clinical symptoms of KM. The questionnaire consists of 44 clinical items measured with a 5-point Likert score (from 1 [none] to 5 [very severe]).

Quality of life: We will use the Korean version of the European Quality of Life-5 Dimensions questionnaire, which has been well validated.

#### 2.7.2 Secondary outcome

Ovarian function testing: Ovarian function testing will be assessed with luteinizing hormone, follicle-stimulating hormone, estradiol, and AMH testing^[13]^ before and after the 3 months of KM treatments. Blood tests will be performed on the 2nd or 3rd day after the menstrual period.

Adverse events: All participants will be required to report any adverse events (AEs) when they occur during the trial at every visit. Each AE will be recorded in the case report form (CRF) by the site investigator and will be assessed for causality.

### 2.8 Data collection and management

We will code the identification of patients. We will also enter data collected at each study visit into a paper CRF with double data entry. Outcome data entered into the database will be verified against the source data on the paper CRFs.

We will encourage patients to complete study and follow-up using text messages and personal calls.

### 2.9 Sample size calculation

Preliminary studies of prospective observational studies require more than 12 participants.^[14,15]^ We calculated the number of patients needed considering the percentage of eligible patients who visit You & Green Korean Medicine Clinic on typical days; the estimated sample size was 40 participants. We will recruit 50 patients considering the dropout rate of 20%.

### 2.10 Statistical analysis

We will analyze categorical and continuous variables with McNemar test and a paired *t* test (in the case of a nonnormal distribution, the Wilcoxon signed rank test will be used), respectively. We will also perform a Chi-squared test or a Fisher exact test for AE rates, the rates of AEs that cause dropout, and the rated of severe AEs.

### 2.11 Ethics and dissemination

The Institutional Review Board of Semyung University approved this study (SMU-IM-190501). Written informed consent will be obtained from all study participants prior to enrollment in the study (Supplement 1). The results will be published in a peer-reviewed journal and will be disseminated electronically and in print regardless of results.

## 3 Discussion

This multicenter observational study was designed to investigate the effects of KM treatment on infertility caused by POR. All KM treatments will be delivered according to the patients’ symptoms and patterns. Although KM treatment or Chinese medicine treatment has been reported to increase the success rate of IVF,^[16,17]^ there is insufficient evidence that KM can improve POR-related infertility. The results of this study will provide us with more reliable research evidence and will provide a better scientific basis for clinicians to rationally choose KM in the treatment of infertile patients with low POR.

## Author contributions

**Conceptualization:** Tae-Young Choi, Ji Hee Jun, Eun Seop Kim, Myeong Soo Lee.

**Data curation:** Tae-Young Choi, Ji Hee Jun, Eun Seop Kim.

**Methodology:** Tae-Young Choi, Ji Hee Jun, Hye Won Lee, Eun Seop Kim, Ho Yeon Go, Sooseong You.

**Resources:** Eun Seop Kim, Myeong Soo Lee.

**Software:** Tae-Young Choi, Ji Hee Jun, Lin Ang.

**Supervision:** Myeong Soo Lee.

**Writing – original draft:** Tae-Young Choi, Ho Yeon Go, Myeong Soo Lee.

**Writing – review & editing:** Ji Hee Jun, Hye Won Lee, Lin Ang, Eun Seop Kim, Sooseong You.

Myeong Soo Lee orcid: 0000-0001-6651-7641.

## Supplementary Material

Supplemental Digital Content
