# Supplementary figures and images for: Korean medicines for poor ovarian reserve in infertility: A protocol for a multicenter observational study
Source: Medicine (Baltimore). 2019 Nov 1;98(44):e17731. doi: 10.1097/MD.0000000000017731 (PMC6946327; doi:10.1097/MD.0000000000017731)

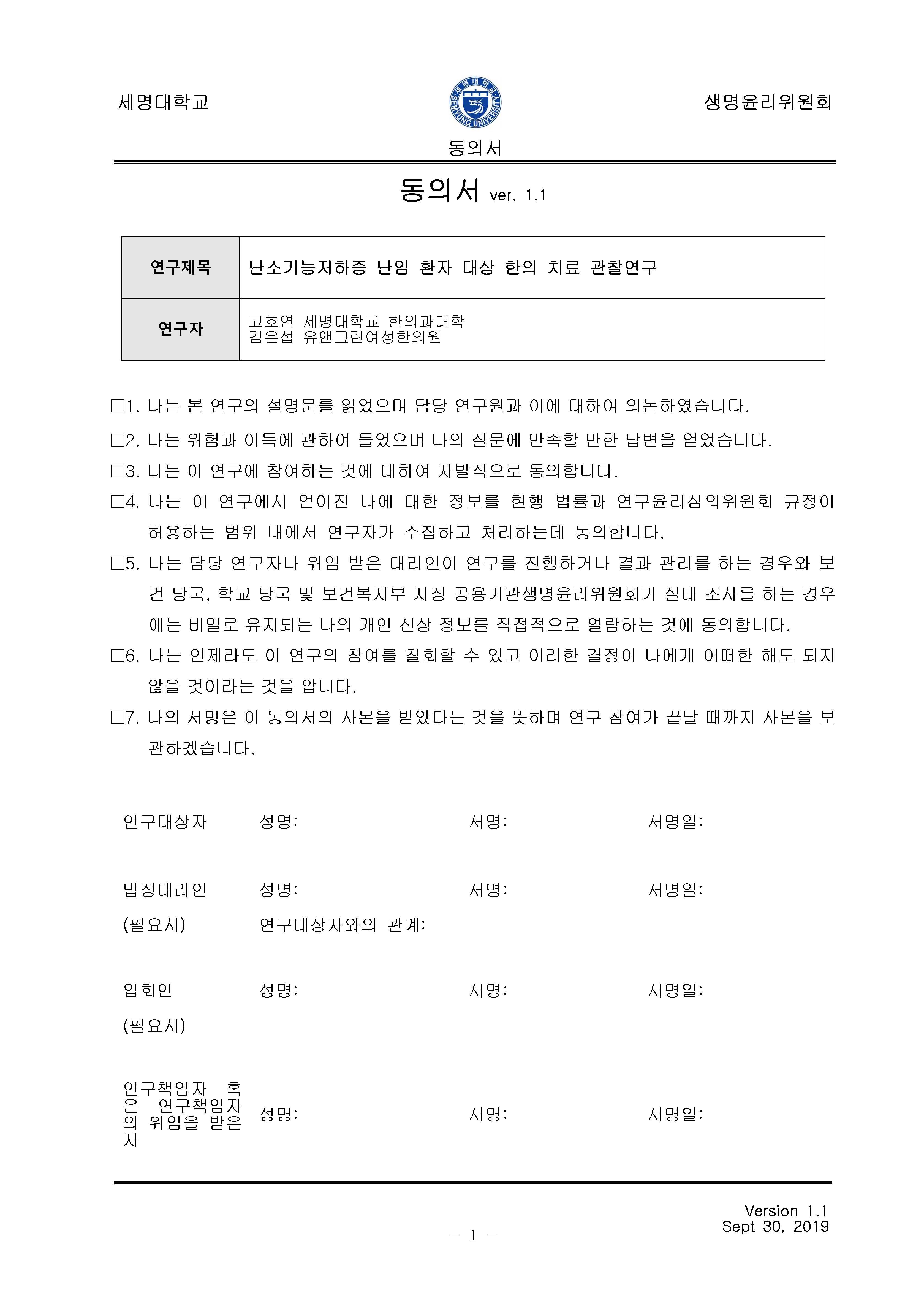

Supplement: Supplemental Digital Content [file medi-98-e17731-s001.jpg]
